# The Role of Metacognition, Intolerance of Uncertainty, and Cognitive‐Attentional Syndrome in Differentiating Fear of Cancer Recurrence

**DOI:** 10.1002/pon.70475

**Published:** 2026-04-29

**Authors:** Wendy W. T. Lam, Danielle W. L. Ng, Ceci Guo, Rachel Ng, Richard Fielding, Chi Chung Foo, Ava Kwong, Siu‐Man Simon Ng, Dacita Suen, Sara Fung, Oi Kwan Chun, Karen K. L. Chan, Wendy W. L. Chan, Amy T. Y. Chang

**Affiliations:** ^1^ Division of Behavioural Sciences Centre for Psycho‐Oncological Research and Training School of Public Health The University of Hong Kong Hong Kong Hong Kong; ^2^ LKS Faculty of Medicine HKU Jockey Club Institute of Cancer Care The University of Hong Kong Hong Kong Hong Kong; ^3^ Department of Surgery The University of Hong Kong Hong Kong Hong Kong; ^4^ Department of Surgery The Chinese University of Hong Kong Hong Kong Hong Kong; ^5^ Department of Surgery Kwong Wah Hospital Hong Kong Hospital Authority Hong Kong Hong Kong; ^6^ Department of Obstetric and Gynaecology The University of Hong Kong Hong Kong Hong Kong; ^7^ Department of Clinical Oncology The University of Hong Kong Hong Kong Hong Kong; ^8^ Comprehensive Oncology Centre Hong Kong Sanatorium & Hospital Hong Kong Hong Kong

**Keywords:** cancer survivorship, cognitive‐attentional syndrome, fear of cancer recurrence, intolerance of uncertainty, metacognition

## Abstract

**Objectives:**

This study tested (1) whether intolerance of uncertainty (IU) and negative metacognitive beliefs independently differentiate fear of cancer recurrence (FCR) status, and (2) whether mediation by cognitive‐attentional syndrome (CAS) elements modify the relationships of IU and metacognitive beliefs with FCR status.

**Methods:**

Baseline data from 384 participants (collected ∼15.6 months post‐diagnosis) pooled from a prospective longitudinal study and an FCR intervention trial were analyzed. Measures included FCRI‐SF (FCR), MCQ‐30 (metacognitive beliefs), IUS‐12 (IU), and CAS‐1 (CAS). FCR status was classified based on the FCRI‐SF scores as non‐clinical (< 13), subclinical (13–21), and clinically significant (≥ 22). Fully adjusted multinomial logistic regression examined direct effects of IU and metacognitive domains on FCR status and indirect effects via CAS.

**Results:**

Compared to the non‐clinical group (*n* = 116), subclinical (*n* = 190) and clinically‐significant (*n* = 78) FCR groups showed greater negative beliefs about worry (OR range 1.297–1.500, *p* < 0.001), higher IU (OR 1.050–1.066, *p* = 0.037), increased use of CAS metacognitive strategies (OR 1.077–1.108, *p* = 0.021‐< 0.001), and less perceived need to control thoughts (OR 0.847–0.857, *p* = 0.003–0.027). Clinically‐significant FCR was additionally associated with poorer cognitive confidence (OR 1.122, *p* = 0.030). CAS partially mediated associations of FCR status with negative beliefs about worry, cognitive confidence, and IU, and fully mediated the association with need to control thoughts.

**Conclusions:**

IU and negative metacognitive beliefs can characterize individuals vulnerable to differentially‐elevated FCR. CAS centrally mediates the relationships between FCR, IU, and metacognition. Cognitive‐behavioral interventions for FCR explicitly targeting CAS should disrupt the impact of IU and metacognitive beliefs on FCR.

## 1 Background

Fear of cancer recurrence (FCR), defined as “fear, worry, or concern about cancer return or progression” [1], is one of the most common challenges for cancer survivors [2] and is among the most frequently reported unmet needs of people living with cancer [3]. A recent meta‐analysis showed that the prevalence of moderate‐to‐high levels of FCR was 59%, whereas 20% demonstrated clinical levels of FCR [2]. Similar to other forms of severe chronic anxiety, high FCR damages long‐term psychosocial functioning, delays recovery, increases the risk of comorbidities, and impairs return to previous roles, exerting growing direct and indirect socio‐economic costs as the number of cancer survivors increases. Several theoretical models of FCR have been developed to investigate the mechanisms contributing to elevated and persistent levels of FCR. Recently, efforts have been made to integrate these models to provide a comprehensive theoretical perspective for the maintenance of a high FCR [4, 5, 6]. These integrated theoretical models of FCR concur that metacognitive beliefs about worry and intolerance of uncertainty (IU) are two factors critically contributing to cognitive vulnerability for, and maintenance of, high FCR levels.

Originally conceptualized within the Self‐Regulatory Executive Function (S‐REF) model, psychological dysfunction is associated with a non‐specific cognitive style termed cognitive attentional syndrome (CAS), characterized by heightened self‐focused attention, rumination, worry, thought suppression, and attentional bias toward threatening stimuli [7, 8]. The activation and persistence of the CAS in response to threatening triggers appears to be due to maladaptive metacognitions about worry [7, 8]. Metacognition consists of two elements: metacognitive knowledge and metacognitive control strategies. Metacognitive knowledge reflects the beliefs that individuals have about their own cognitions and internal states (i.e., beliefs about the significance of particular thinking processes), whereas metacognitive control strategies embody the information that individuals hold about coping strategies (such as their effectiveness) used to regulate their cognitive activity. The concept of maladaptive metacognition about worry has been studied using the Metacognition Questionnaire (MCQ‐30) [7] which assesses five domains of metacognitive thoughts: (1) positive beliefs about worry, (2) negative beliefs about thoughts concerning uncontrollability danger, (3) cognitive confidence in attention and memory, (4) negative beliefs concerning the consequences of not controlling thoughts, and (5) the tendency to focus attention on thought processes. In the context of FCR, previous studies have shown that certain domains of metacognitive beliefs about worry are associated with heightened FCR [9, 10, 11, 12, 13, 14, 15]. For instance, positive beliefs about worry (e.g., worrying helps me prepare in case cancer recurs) may reinforce ruminative worry as an emotional‐focussed coping strategy, leading to increased FCR. Similarly, counterproductive thoughts and/or emotion suppression may arise from negative beliefs about worry (e.g., worrying causes cancer to spread), generating intrusive and catastrophic thinking [16] and promoting heightened attentional bias toward threat‐related information, as well as more intense future negative emotions [17], thus exacerbating and maintaining FCR [10]. While the roles of attentional bias and maladaptive coping strategies (features of CAS) in FCR have been previously investigated, the mediating effect of CAS on the relationship between metacognition and FCR has rarely been tested. A recent longitudinal study examined whether metacognitive beliefs differentiated FCR trajectories and tested whether CAS (attentional bias, intrusive thoughts, and avoidance) mediated the relationship between metacognitive beliefs and FCR trajectories among cancer survivors [15]. The effect of negative beliefs about worry on FCR was partially mediated by avoidance and fully mediated by intrusive thoughts.

Intolerance of uncertainty (IU), which refers to beliefs about the importance of having certainty in life and one's ability to manage unpredictability or ambiguity [18] has been linked to anxiety‐related disorders [19, 20]. According to the IU model, IU is defined as a cognitive bias that affects how an individual perceives, interprets, and responds to uncertain situations [21]. It has been conceptualized with two key characteristics: prospective and inhibitory IU [18]. Prospective IU is the information‐seeking dimension, characterized by a desire for predictivity and knowing what the future holds, being anxious about future uncertain events, and engaging in information‐seeking to increase the sense of certainty, whereas inhibitory IU is the avoidance dimension, characterized by avoidance and paralysis in the face of uncertainty [18]. Research suggests that IU plays an important role in the acquisition and maintenance of worries, as the persistence of IU may result in biased information processing in the context of ambiguity, threatening interpretations of uncertainty, and negatively reinforcing certainty‐seeking behaviors. Given that cancer survivors face uncertainty regarding cancer recurrence, individuals' ability to cope with this uncertainty may affect FCR. In contrast to the role of metacognitive beliefs about worry in FCR, the findings regarding the role of IU in FCR are mixed. Two earlier studies found no association between IU and FCR [5, 14], whereas four recent studies found a positive association [13, 22, 23, 24]. With the exception of one study focusing on patients newly diagnosed with cancer [22] and one limited to the early survivorship period [24], other studies included patients who were long‐term cancer survivors. However, IU may become less relevant over time as the risk of cancer recurrence is substantively lower 5 years after the initial treatment [13]. Interestingly, there is evidence that IU influences the symptoms of generalized anxiety disorder by increasing repetitive negative thoughts, or rumination, to control feelings of uncertainty and anxiety about future events [13, 18, 20]. Rumination has been reported to fully mediate the relationship between IU and major depression disorder [25]. These findings raise the possibility of an interrelationship between IU, CAS, and emotional disturbance. The mediating effect of CAS on the relationship between IU and FCR has not yet been investigated. To date, no study has concurrently examined IU, negative metacognitive beliefs about worry, CAS and FCR. Moreover, no prior research has investigated whether differences in metacognitive beliefs regarding worry and IU exist between normal, subclinical, and clinical FCR status, as existing studies have primarily explored their impacts on levels of FCR. Therefore, we examined whether IU, negative metacognitive beliefs about worry, and CAS differentiate FCR status. Specifically, we tested (1) the role of IU and negative metacognitive beliefs about worry in independently differentiating FCR status, (2) the mediating effect of CAS on the relationship between IU and FCR status, and (3) the mediating effect of CAS on the relationship between metacognitive beliefs about worry and FCR status. We hypothesized that (1) IU and negative metacognitive beliefs about worry would independently differentiate FCR status, (2) CAS would mediate the relationship between negative metacognitive beliefs about worry and FCR status, and (3) CAS would mediate the relationship between IU and FCR status.

## 2 Methods

### 2.1 Participants and Procedure

Participants were drawn from two studies, one a prospective longitudinal study and the other a FCR intervention study [26, 27]. In this 12‐month longitudinal study, we recruited Cantonese‐ or Mandarin‐speaking Chinese patients who completed treatment within 18 months for non‐metastatic breast cancer or colorectal cancer. The exclusion criteria were non‐Chinese ethnicity, metastatic cancer, current diagnosis of depression or psychosis, and language difficulties or an intellectual disability. Recruitment was conducted at two government‐funded breast cancer centers and two colorectal cancer centers in Hong Kong. Ethical approval was obtained from the HKU/Hospital Authority (reference no. UW21‐164). All eligible patients awaiting follow‐up medical consultation at the cancer centers were approached by trained research assistants. After explaining the participants' rights and the safeguards for anonymity, willing participants were asked to complete the baseline questionnaire during the initial contact, and then at 3‐month, 6‐month, and 12‐month from baseline. Only baseline data from 209 participants were used in this study. To capture patients with subclinical and clinical FCR status, we included participants from the fear of cancer recurrence intervention study [26, 27]. Recruitment for the intervention study was conducted at six medical centers in Hong Kong that offered breast, colorectal, or gynecological cancer care. Ethical approval was obtained from the HKU/Hospital Authority (reference no. UW19‐183). The sample selection criteria were consistent with those employed in the longitudinal study. However, in the trial study, we exclusively included patients who scored 13 or higher on the Fear of Cancer Recurrence Inventory‐Short Form (FCRI‐SF), indicating subclinical‐to‐clinical levels of FCR [28]. Only baseline data from 175 participants were used in this study. Thus, 384 participants were included in the current study. Informed consents were obtained from all participants prior to data collection.

### 2.2 Measures

Metacognitive beliefs about worry were assessed using the Metacognitions Questionnaire‐30 (MCQ‐30) [7]. The MCQ‐30 is a 30‐item measure that assesses individual differences in metacognitive beliefs, judgements, and monitoring tendencies. It consists of five domains: (1) positive beliefs about worry, (2) negative beliefs about thoughts concerning uncontrollability and danger, (3) cognitive confidence, (4) cognitive self‐consciousness, and (5) the need to control thoughts. All items were rated on a four‐point Likert scale ranging from 1 (do not agree) to 4 (agree very much). Higher scores in each domain indicate higher levels of maladaptive metacognition regarding worry. The MCQ‐30 has demonstrated good internal consistency (Cronbach's *α* ranged from 0.72 to 0.93), convergent validity, and test‐retest reliability [7]. The Chinese version of the MCQ‐30 also demonstrated good internal consistency (Cronbach's *α* = 0.83–0.87) [11, 15]. Domain scores were used in the present study given evidence indicates that metacognitive domains play distinct roles in contributing to FCR. Hence, examining each domain separately allows for a more precise understanding of how distinct metacognitive components contribute to FCR [9, 10, 11, 12, 13, 14, 15].

Intolerance of Uncertainty was assessed using the 12‐item Intolerance of Uncertainty Scale‐Short Form (IUS‐12) [29]. The IUS‐12 comprises two subscales: prospective IU (7items) and inhibitory IU (5items). Prospective IU assesses fear and anxiety related to cognitive anticipation, while Inhibitory IU assesses the degree to which uncertainty inhibits action or experience. Items were scored on a Likert scale ranging from 1 (not at all characteristic of me) to five (entirely characteristic of me), with higher scores indicating greater intolerance of uncertainty. The IUS‐12 has strong internal consistency (Cronbach's *α* = 0.91) and convergent validity [29]. In the current study, the Chinese version of the IUS‐12 demonstrated good internal reliability (Cronbach's *α* = 0.92).

Cognitive‐attentional syndrome was assessed using the Cognitive‐attentional syndrome‐1 (CAS‐1) questionnaire [30]. The CAS‐1 is a 16‐items measure that assesses the frequency of engaging in rumination, threat monitoring, and coping behaviors, as well as the level of metacognitive beliefs. With the exception of items measuring metacognitive beliefs, each item was rated on a 9‐point Likert scale ranging from 0 to 8. For items measuring metacognitive beliefs, each item was rated on a scale of 0–100. Recent evidence shows that the CAS‐I consists of three subscales measuring metacognitive strategies, positive and negative metacognitive beliefs [30]. Higher scores indicate higher levels of CAS activation. The CAS‐1 demonstrated good internal consistency (Cronbach's *α* 0.83–0.91) and convergent validity [30]. To minimize any multicollinearity issues with the MCQ‐30, which measures metacognitive beliefs, only the subscale of metacognitive strategies assessing rumination, threat monitoring, and avoidance or reassurance coping behaviors was used in the current study. The Chinese version of the CAS‐1 subscale of metacognitive strategies demonstrated good internal reliability (Cronbach's *α* 0.88).

Fear of cancer recurrence was assessed using the 42‐item Fear of Cancer Recurrence Inventory (FCRI) [28]. The FCRI measures seven dimensions of FCR: triggers, severity, psychological distress, functional impairment, reassurance, insights, and coping strategies. All items are rated on a five‐point Likert scale ranging from 0 (not at all or never) to 4 (a great deal or all the time). Higher scores indicate higher FCR levels. In the current study, the nine‐item severity subscale (FCRI‐SF), with scores ranging from 0 to 36, was used to categorise FCR status as follows non‐clinical FCR with scores of < 13; subclinical FCR with scores of 13–21; and clinically‐significant FCR with scores ≥ 22 [28]. In the current study, the Chinese version of both FCRI and FCR‐SF [11, 15] demonstrated good internal consistency (Cronbach's *α* 0.96 and 0.85, respectively).

### 2.3 Demographics and Medical Characteristics

Participants' self‐reported socio‐demographic (age, gender, marital status, education, occupation, and household income) data were collected at the baseline interview in both studies, and clinical data were extracted from medical records.

### 2.4 Statistical Methods

Standard descriptive analyses were used to assess and compare the sample characteristics according to the FCR status. Univariate analyses examined the associations between FCR status and each demographic and clinical variable, with associations exceeding *p* < 0.05, and were included in the subsequent multivariate analyses. Next, multinomial logistic regression analysis was used to test if IU, and negative metacognitive beliefs independently differentiated the FCR status. Variance inflation factor (VIF) scores were used to assess multicollinearity, with VIF values below five indicating minimal concern [31]. All the VIF scores for the study variables were ≤ 2.56, suggesting that multicollinearity was insignificant. To test the mediating effect of CAS metacognitive strategies on the relationship between IU and FCR status, as well as on the relationship between metacognition and FCR status, Hayes' PROCESS macro in SPSS was used [32].

## 3 Results

The current study comprised a total sample of 384 participants, categorized using the FCRI‐SF as follows: 116 individuals (30%) were defined as having non‐clinical FCR, 190 individuals (50%) as subclinical FCR, and 78 individuals (20%) as clinically‐significant FCR. All non‐clinical FCR participants were from the prospective longitudinal study sample, whereas only 40% (*n* = 76) of subclinical and 22% (*n* = 17) of clinically‐significant FCR came from this sample; the remaining 60% (*n* = 114) and 78% (*n* = 61), respectively, were from the FCR intervention sample [26, 27]. The sample characteristics of subclinical and clinically‐significant FCR cases did not differ significantly between the two studies. Table 1 compares the sample characteristics according to the FCR status. Compared with individuals with non‐clinical FCR, those with subclinical FCR or clinically‐significant FCR were more likely to be younger (*F* = 31.86, df2, *p* < 0.001), female (*χ* ^2^ = 25.84, *p* < 0.001), have higher educational attainment (*χ* ^2^ = 15.00, *p* = 0.001), higher household income (*χ* ^2^ = 23.75, *p* = 0.008), and diagnosed with breast or gynecological cancer (*χ* ^2^ = 62.09, *p* < 0.001).

**TABLE 1:** Sample characteristics (*n* = 384).

	Non‐clinical FCR (*N* = 116)	Subclinical FCR (*N* = 190)	Clinically‐significant FCR (*N* = 78)	*p*‐value
Age (years)	64.32 (SD 9.02)	55.86 (SD 10.49)	54.58 (SD 10.31)	< 0.001
Gender				< 0.001
Female	78 (67.2%)	169 (88.9%)	69 (88.5%)	
Male	38 (32.8%)	21 (11.1%)	9 (11.5%)	
Marital status				NS
Married	83 (71.6%)	122 (64.2%)	44 (56.4%)	
Single/divorced/widowed	33 (28.4%)	68 (35.8%)	33 (43.6%)	
Education level				
No formal/Primary education	30 (25.9%)	20 (10.5%)	8 (10.3%)	0.001
Secondary/Tertiary education	86 (74.1%)	170 (89.5%)	70 (89.7%)	
Occupation				NS
Employed	40 (34.5%)	89 (46.8%)	36 (46.2%)	
Retired	53 (45.7%)	50 (26.3%)	17 (21.8%)	
Housewife	16 (13.8%)	32 (16.8%)	12 (15.4%)	
Unemployed	7 (6%)	19 (10.0%)	13 (16.7%)	
Monthly household income (HK$)				0.008
≤ 10,000	38 (40.0%)	45 (24.8%)	27.4 (11.3%)	
10,001–30,000	30 (31.5%)	47 (30.0%)	26 (35.6%)	
30,001–40,000	11 (11.6%)	23 (13.2%)	10 (13.7%)	
> 40,000	16 (16.8%)	59 (33.9%)	17 (23.3%)	
Cancer type				< 0.001
Breast cancer	57 (49.1%)	125 (65.8%)	44 (56.4%)	
Colorectal cancer	55 (47.4%)	35 (18.4%)	13 (16.75%)	
Gynecological cancer	0 (0%)	28 (14.7%)	20 (25.6%)	
Lung cancer	1 (0.9%)	2 (1.1%)	1 (1.3%)	
Time since diagnosis (months)	15.66 (6.61)	15.40 (7.37)	16.17 (7.41)	NS
Currently on active treatment (Yes)	33 (28.4%)	75 (39.5%)	24 (30.8%)	NS

Abbreviation: NS: Non‐significant.

Analysis of Variance (ANOVA) revealed significant differences among the FCR groups concerning metacognition, IU, and CAS (Table 2). Multinomial logistic regression analysis was conducted to test the role of IU, negative metacognitive beliefs, and CAS metacognitive strategies in independently differentiating the FCR status, adjusting for the effect of significant demographic and clinical covariates. Only age was a significant covariate and was therefore retained in the final regression model (Table 3). Compared to the non‐clinical FCR group (reference), the subclinical and clinically‐significant FCR groups were younger (odds ratio (OR) = 0.926, 95% confidence interval (CI) 0.895–0.959; OR = 0.922, 95% CI 0.883–0.963, respectively) and reported greater negative beliefs about worry (OR = 1.297, 95% CI 1.135–1.481; OR = 1.500, 95% CI 1.280–1.758, respectively), perceived less need to control thoughts (OR = 0.847, 95% CI 0.795–0.946; OR = 0.857, 95% CI 0.747–0.983, respectively), reported greater IU (OR = 1.050, 95% CI 1.003–1.100; OR = 1.066, 95% CI 1.004–1.132, respectively) and greater use of CAS metacognitive strategies (OR = 1.077, 95% CI 1.034–1.123; OR = 1.108, 95% CI 1.055–1.163, respectively). Furthermore, the clinically‐significant FCR group also reported a greater lack of cognitive confidence (OR = 1.122, 95% CI 1.011–1.245) than the non‐clinical FCR group. The final multinomial regression model accounted for 50% (Cox and Snell *R* ^2^) of the observed variance.

**TABLE 2:** Scores of Metacognitions, Intolerance of uncertainty and cognitive attentional syndrome by FCR status.

	Non‐clinical FCR (*N* = 116) Mean (SD)	Subclinical FCR (*N* = 190) Mean (SD)	Clinically‐significant FCR (*N* = 78) Mean (SD)	*p*‐value
Metacognition (MCQ‐30)	48.15 (10.59)	58.68 (12.05)	68.99 (13.09)	< 0.001
MCQ‐30 positive beliefs about worry	7.12 (2.77)	8.39 (3.08)	9.46 (3.83)	< 0.001
MCQ‐30 negative beliefs about worry	7.65 (2.60)	11.78 (3.98)	15.64 (4.19)	< 0.001
MCQ‐30 need to control thoughts	11.08 (3.58)	11.67 (3.60)	13.71 (3.55)	< 0.001
MCQ‐30 cognitive confidence	9.50 (3.65)	12.03 (4.24)	14.72 (4.58)	< 0.001
MCQ‐30 cognitive self‐consciousness	12.79 (4.85)	14.82 (4.09)	15.46 (3.66)	< 0.001
Intolerance of uncertainty (IU)	22.79 (8.97)	31.55 (8.96)	30.32 (10.64)	< 0.001
Cognitive‐attentional syndrome (CAS) metacognitive strategies	5.67 (8.09)	18.06 (11.74)	26.76 (10.98)	< 0.001

**TABLE 3:** Multinomial logistic regression of metacognition, Intolerance of uncertainty and Cognitive attentional syndrome on FCR status (Non‐clinical FCR as reference).

Reference: Non‐clinical FCR group	Subclinical FCR group			Clinically‐significant FCR group		
Covariates	Odds ratio (95% CI)	SE	*p* value	Odds ratio (95% CI)	SE	*p* value
MCQ‐30 positive beliefs about worry	1.020 (0.898, 1.159)	0.065	NS	1.045 (0.902, 1.211)	0.075	NS
MCQ‐30 negative beliefs about worry	1.297 (1.135, 1.481)	0.068	< 0.001	1.500 (1.280, 1.757)	0.081	< 0.001
MCQ‐30 cognitive self‐consciousness	0.985 (0.909, 1.068)	0.041	NS	0.938 (0.842, 1.046)	0.05	NS
MCQ‐30 lack of cognitive confidence	1.065 (0.979, 1.158)	0.043	NS	1.122 (1.011, 1.245)	0.053	0.030
MCQ‐30 need to control thoughts	0.847 (0.759, 0.946)	0.056	0.003	0.857 (0.747, 0.983)	0.070	0.027
Intolerance of uncertainty (IU)	1.050 (1.003, 1.100)	0.024	0.037	1.066 (1.004, 1.132)	0.031	0.037
CAS metacognitive strategies	1.077 (1.034, 1.123)	0.021	< 0.001	1.108 (1.055, 1.163)	0.025	< 0.001
Age	0.926 (0.895, 0.959)	0.018	< 0.001	0.922 (0.883, 0.963)	0.022	< 0.001

Abbreviation: NS: Non‐significant.

Next, we tested the mediating effect of CAS metacognitive strategies on the relationship between metacognition about worry and FCR status, as well as between IU and FCR status (Table 4). First, we tested the mediating effect of CAS metacognitive strategies on the relationship between MCQ‐30 negative beliefs about worry and FCR status. Adjusting for the effect of age, MCQ‐30 negative beliefs about worry was positively associated with CAS metacognitive strategies (*β* = 1.75, *p* < 0.001) and with FCR status (non‐clinical FCR vs. subclinical or clinical‐significant FCR) (*β* = 0.43, *p* < 0.001). CAS metacognitive strategies, the putative mediator, was positively associated with FCR status (*β* = 0.080, *p* < 0.001). The indirect effect was tested using non‐parametric bootstrapping [32]. The mediation analysis revealed a significant indirect effect of MCQ‐30 negative beliefs about worry on FCR status through CAS metacognitive strategies (*β* = 0.14, SE 0.037, 95% CI 0.078, 0.225). After controlling for the mediator, CAS metacognitive strategies, MCQ negative beliefs about worry on FCR status retained a reduced but significant direct effect (*β* = 0.29, *p* < 0.001), suggesting that CAS metacognitive strategies partially mediate the association between MCQ negative beliefs about worry and FCR status (Supporting Information S1).

**TABLE 4:** Mediation analysis of cognitive attentional syndrome (CAS) on the relationship of metacognition and intolerance of uncertainty (IU) on FCR status (non‐clinical FCR vs. subclinical/clinical‐significant FCR).

	MCQ‐30 negative beliefs about worry				MCQ‐30 need to control thoughts				MCQ‐30 cognitive confidence				IU			
	*β*	SE	*p*‐value	95% CI	*β*	SE	*p*‐value	95% CI	*β*	SE	*p*‐value	95% CI	*β*	SE	*p*‐value	95% CI
CAS	1.752	0.109	< 0.001	1.535, 1.968	1.425	0.155	< 0.001	1.119, 1.731	1.131	0.129	< 0.001	0.876, 1.385	0.746	0.048	< 0.001	0.652, 0.839
FCR status	0.428	0.038	< 0.001	1.355, 1.737	−0.102	0.051	0.044	0.818, 0.997	0.084	0.041	0.042	1.003, 1.180	0.123	0.017	< 0.001	1.096, 1.169
CAS → FCR status	0.080	0.019	< 0.001	0.043, 0.118	0.143	0.018	< 0.001	0.107, 0.178	0.123	0.017	< 0.001	0.089, 0.158	0.104	0.019	< 0.001	0.068, 0.140
FCR status adjusting for the indirect effect of CAS	0.290	0.059	< 0.001	0.174, 0.407	−0.046	0.042	0.284	−0.129, 0.038	0.114	0.039	0.003	0.038, 0.189	0.066	0.019	0.0006	0.028, 0.103

Next, we tested the mediating effect of CAS metacognitive strategies on the relationship between the MCQ perceived need to control thoughts and FCR status. Adjusting for the effect of age, MCQ‐30 perceived need to control thoughts was positively associated with CAS metacognitive strategies (*β* = 1.42, *p* < 0.001) and negatively associated with FCR status (*β* = −0.102, *p* < 0.001). CAS metacognitive strategies, the putative mediator, was positively associated with FCR status (*β* = 0.143, *p* < 0.001). The mediation analysis revealed a significant indirect effect of MCQ perceived need to control thoughts on FCR status through CAS metacognitive strategies (*β* = 0.20, SE 0.041, 95% CI 0.139, 0.297). After controlling for the mediator, CAS metacognitive strategies, the direct effect of MCQ perceived need to control thoughts on FCR status was insignificant (*β* = −0.046, *p* = 0.284), suggesting full mediation by CAS metacognitive strategies (Supporting Information S1).

Adjusting for the effect of age, MCQ lack of cognitive confidence was positively associated with CAS metacognitive strategies (*β* = 1.13, *p* < 0.001) and with FCR status (*β* = 0.084, *p* = 0.042). CAS metacognitive strategies, the putative mediator, was positively associated with FCR status (*β* = 0.123, *p* < 0.001). The mediation analysis revealed a significant indirect effect of MCQ lack of cognitive confidence on FCR status through CAS metacognitive strategies (*β* = 0.14, SE 0.029, 95% CI 0.093, 0.207). Upon adjusting for the mediator, CAS metacognitive strategies, the direct effect of MCQ lack of cognitive confidence on FCR status remained as significant, though diminished (*β* = 0.114, *p* = 0.003), suggesting that CAS metacognitive strategies partially mediate the relationship (Supporting Information S1).

Finally, we tested the mediating effect of CAS metacognitive strategies on the relationship between IU and FCR status. Adjusting for the effect of age, IU was positively associated with CAS metacognitive strategies (*β* = 0.746, *p* < 0.001) and with FCR status (*β* = 0.124, *p* < 0.001). CAS metacognitive strategies, the putative mediator, was positively associated with FCR status (*β* = 0.104, *p* < 0.001). The mediation analysis revealed a significant indirect effect of IU on FCR status through CAS metacognitive strategies (*β* = 0.078, SE 0.016, 95% CI 0.051, 0.113). When accounting for the mediator, CAS metacognitive strategies, the direct effect of IU on FCR status remained, albeit reduced, with a significant direct effect (*β* = 0.066, *p* = 0.0006). This indicates that CAS metacognitive strategies partially mediate the relationship (Supporting Information S1).

## 4 Discussion

This study tested the role of negative metacognitive beliefs about worry and IU in independently differentiating FCR status and the mediating effect of CAS on the relationship between metacognitive beliefs about worry and IU on FCR status. The hypothesis that negative metacognitive beliefs about worry and IU would differentiate FCR status was supported. The hypothesis that CAS mediates the relationship between metacognitive beliefs about worry and IU on FCR status was also supported.

Our study provides evidence that metacognitive beliefs about worry represent a multidomain construct, with each domain exerting a distinct influence on FCR. Consistent with prior studies [9, 11, 12, 15], our findings showed that individuals with subclinical or clinically‐significant FCR reported greater MCQ‐30 negative metacognitive beliefs about worry than those with non‐clinical FCR. Furthermore, our study revealed that the role of MCQ‐30 negative beliefs about worry in differentiating FCR status was partially mediated by CAS, which encompasses rumination, threat monitoring, and maladaptive coping behaviors. Our findings suggest that individuals with subclinical or clinically‐significant FCR are likely to worry about worry, with these negative beliefs about worry activating the CAS, which in turn promotes FCR. Interestingly, the MCQ‐30 perceived need to control thoughts emerged as another metacognitive domain relevant in differentiating FCR status, but uniquely, its effect was solely through CAS. The findings of the relationship between the MCQ‐30 perceived need to control thoughts and FCR in previous studies have been mixed. An earlier FCR study reported a positive correlation between the MCQ‐30 perceived need to control thoughts and the level of FCR [9], but more recent studies have not observed this association [10, 12, 13, 15]. With the exception of one study [24], previous studies did not assess whether differences in metacognitive belief levels exist across non‐clinical, subclinical, and clinically‐significant samples. Our findings are consistent with previous meta‐analysis studies comparing clinical and non‐clinical samples of psychiatric diagnoses testing the S‐REF model, in which MCQ‐30 negative metacognitive beliefs about worry and the MCQ‐30 need to control thoughts are the two strongest factors in maintaining psychological distress [33, 34]. Our findings also support the S‐REF model, which emphasizes the role of CAS in elucidating the relationship between maladaptive metacognition and psychological dysfunction [7, 8, 11].

Another metacognitive domain, cognitive confidence, also played a distinctive role. Individuals with clinically‐significant FCR reported poorer MCQ‐30 cognitive confidence in memory than those with non‐clinical FCR. This concurs with the findings of our previous longitudinal study, in which individuals with high‐stable FCR levels reported poorer MCQ‐30 cognitive confidence than those with low‐stable FCR [15]. The role of lack of cognitive confidence in differentiating FCR status was partially mediated by CAS. Poor cognitive confidence creates self‐doubt and uncertainty, which may, in turn, promote intrusive thoughts, threat monitoring, and maladaptive coping behaviors, resulting in heightened FCR [15].

Consistent with studies focusing on the early survivorship phase [22, 24], our findings showed that individuals with subclinical or clinically‐significant FCR reported greater IU than those with non‐clinical FCR. Previous studies on long‐term cancer survivorship have not demonstrated consistent results regarding the relationship between IU and FCR. These findings may reflect that IU is more relevant in the early survivorship phase, when there is greater uncertainty across multiple aspects, including managing treatment side/late effects and returning to normalcy [24]. Our study, the first to examine the mediating effect of CAS, confirmed that the role of IU in distinguishing FCR status was partially mediated by CAS. Higher levels of IU were observed among individuals with subclinical or clinically‐significant FCR, and higher levels of IU were associated with CAS, which resulted in heightened FCR. Our findings suggest that individuals who struggle with uncertainty may rely more heavily on maladaptive coping and attentional strategies, such as rumination, threat monitoring, or avoidance, to manage their discomfort with the unknown. These strategies reinforce FCR by increasing attentional focus on potential signs of recurrence and amplifying worries about future health threats.

The findings of this study highlight how both the S‐REF and IU models offer complementary explanations for the mechanisms underlying FCR. Consistent with the S‐REF framework, maladaptive metacognitive beliefs, particularly negative beliefs about worry, perceived need to control thoughts, and low cognitive confidence, differentiated FCR status and largely exerted their effects through the activation of the CAS. Similarly, IU emerged as a significant cognitive vulnerability for those with subclinical or clinically‐significant FCR, especially through its effect on CAS. Together, our findings suggest that metacognitive vulnerabilities and difficulty tolerating uncertainty converge on the same maladaptive cognitive‐attentional pathway, reinforcing the centrality of the CAS as a shared mechanism driving heightened FCR.

### 4.1 Clinical Implications

Our findings suggest that clinical interventions for FCR should target maladaptive metacognitive beliefs and IU. A recent meta‐analysis showed the significant and lasting effect of cognitive‐behavioral therapies (CBT) on improving FCR among cancer survivors and showed greater FCR improvement for contemporary CBTs with a cognitive process focus targeting metacognition [35]. However, existing interventions have paid limited attention to structured strategies for managing uncertainty, despite the current study showing that IU significantly differentiates FCR status and contributes to heightened FCR through its effect on CAS. Furthermore, our findings demonstrate that the CAS plays a central role in linking both negative metacognitive beliefs about worry and IU with FCR status, with the CAS partially or fully mediating these associations. This suggests that interventions may be strengthened by incorporating modules that directly manage CAS, alongside techniques that modify metacognitive beliefs and enhance tolerance of uncertainty. Integrating these components may further optimize the effectiveness of FCR interventions by targeting the shared cognitive‐attentional pathway through which maladaptive metacognitive beliefs about worry and IU amplify FCR.

### 4.2 Study Limitations

This study has several limitations. The current study was based on cross‐sectional data; therefore, causal relationships could not be determined. The over‐representation of the females in the sample (82%) and the diagnosis of breast cancer (59%) prevented us from examining the effect of gender and cancer type on FCR status. Furthermore, to obtain sufficient participants with subclinical and clinically significant FCR levels, we used two clinical samples, one from a prospective longitudinal study and the other from a FCR intervention study. While both samples were recruited from similar clinical settings and no difference in demographic or clinical characteristics among those with subclinical or clinically significant FCR were observed across the two samples, results nevertheless need to be interpreted with caution, as combining samples from two distinct studies may limit the generalizability of the findings despite comparable demographic and clinical characteristics. Finally, while our regression model explained 50% of the variance in differentiating FCR status, the present study only examined the role of metacognitive beliefs, IU, and CAS. Other factors, such as triggers, perceived risk of recurrence, and illness uncertainty, proposed in recent blended theoretical models of FCR [5, 14], may also be important contributing factors that need to be tested in future studies.

## 5 Conclusion

This study provides partial validation of the S‐REF model in the context of FCR. It demonstrates the direct effects of maladaptive metacognitions and CAS on the development of FCR, as well as the mediating role of CAS in the association between metacognitions and FCR. Additionally, IU was found to contribute to elevated FCR by amplifying CAS. Cancer survivors with higher levels of maladaptive metacognitions and IU were more likely to engage in rumination, threat monitoring, and unhelpful coping behaviors, which in turn intensified FCR.

The identification of these underlying mechanisms informs the refinement of widely used, evidence‐based metacognition‐focused interventions. Specifically, incorporating strategies to address uncertainty management may enhance the effectiveness of FCR interventions and improve outcomes in survivorship care.

## Author Contributions

W.W.T. Lam conceptualized the study, acquired funding, conducted data analysis, and drafted the original manuscript. D.W.L. Ng contributed to data analysis and manuscript writing. C. Guo and R. Ng were responsible for data collection. R Fielding reviewed and edited the manuscript. All other authors participated in data collection as site coordinators. All authors reviewed and approved the final manuscript.

## Conflicts of Interest

The authors declare no conflicts of interest.

## Supporting information


**Figure S1:** (A) Model showing a mediation role of CAS metacognitive strategies in the relationship between MCQ‐30 negative beliefs about worry and FCR status (non‐clinical FCR Vs subclinical/clinical‐significant FCR). (B) Model showing a mediation role of CAS metacognitive strategies in the relationship between MCQ‐30 need to control thoughts and FCR status (non‐clinical FCR Vs subclinical/clinical‐significant FCR). (C) Model showing a mediation role of CAS metacognitive strategies in the relationship between MCQ‐30 cognitive confidence and FCR status (non‐clinical FCR Vs subclinical/clinical‐significant FCR). (D) Model showing a mediation role of CAS metacognitive strategies in the relationship between Intolerance of uncertainty (IU) and FCR status (non‐clinical FCR Vs subclinical/clinical‐significant FCR).

## Data Availability

The data that support the findings of this study are available from the corresponding author upon reasonable request.

## References

[1] S. Lebel , G. Ozakinci , G. Humphris , et al., “From Normal Response to Clinical Problem: Definition and Clinical Features of Fear of Cancer Recurrence,” Supportive Care in Cancer 24, no. 8 (2016): 3265–3268, 10.1007/s00520-016-3272-5. 27169703

[2] Y. L. Luigjes‐Huizer , N. M. Tauber , G. Humphris , et al., “What Is the Prevalence of Fear of Cancer Recurrence in Cancer Survivors and Patients? A Systematic Review and Individual Participant Data Meta‐Analysis,” Psycho‐Oncology 31, no. 6 (2022): 879–892, 10.1002/pon.5921. 35388525 PMC9321869

[3] J. Armes , M. Crowe , L. Colbourne , et al., “Patients' Supportive Care Needs Beyond the End of Cancer Treatment: A Prospective, Longitudinal Survey,” Journal of Clinical Oncology 27, no. 36 (2009): 6172–6179, 10.1200/jco.2009.22.5151. 19884548

[4] L. Curran , L. Sharpe , and P. Butow , “Anxiety in the Context of Cancer: A Systematic Review and Development of an Integrated Model,” Clinical Psychology Review 56 (2017): 40–54, 10.1016/j.cpr.2017.06.003. 28686905

[5] S. Lebel , C. Maheu , C. Tomei , et al., “Towards the Validation of a New, Blended Theoretical Model of Fear of Cancer Recurrence,” Psycho‐Oncology 27, no. 11 (2018): 2594–2601, 10.1002/pon.4880. 30180279

[6] L. E. Simonelli , S. D. Siegel , and N. M. Duffy , “Fear of Cancer Recurrence: A Theoretical Review and Its Relevance for Clinical Presentation and Management,” Psycho‐Oncology 26, no. 10 (2017): 1444–1454, 10.1002/pon.4168. 27246348

[7] A. Wells and G. Matthews , “Modelling Cognition in Emotional Disorder: The S‐REF Model,” Behaviour Research and Therapy 34, no. 11–12 (1996): 881–888, 10.1016/s0005-7967(96)00050-2. 8990539

[8] M. M. Spada , G. A. Georgiou , and A. Wells , “The Relationship Among Metacognitions, Attentional Control, and State Anxiety,” Cognitive Behaviour Therapy 39, no. 1 (2010): 64–71, 10.1080/16506070902991791. 19735025

[9] B. Thewes , M. L. Bell , and P. Butow , “Fear of Cancer Recurrence in Young Early‐Stage Breast Cancer Survivors: The Role of Metacognitive Style and Disease‐related Factors,” Psycho‐Oncology 22, no. 9 (2013): 2059–2063, 10.1002/pon.3252. 23408595

[10] P. Butow , S. Kelly , B. Thewes , G. Hruby , L. Sharpe , and J. Beith , “Attentional Bias and Metacognitions in Cancer Survivors With High Fear of Cancer Recurrence,” Psycho‐Oncology 24, no. 4 (2015): 416–423, 10.1002/pon.3659. 25156065

[11] D. W. L. Ng , A. Kwong , D. Suen , et al., “Fear of Cancer Recurrence Among Chinese Cancer Survivors: Prevalence and Associations With Metacognition and Neuroticism,” Psycho‐Oncology 28, no. 6 (2019): 1243–1251, 10.1002/pon.5073. 30932279

[12] A. B. Smith , L. Sharpe , B. Thewes , et al., “Medical, Demographic and Psychological Correlates of Fear of Cancer Recurrence (FCR) Morbidity in Breast, Colorectal and Melanoma Cancer Survivors With Probable Clinically Significant FCR Seeking Psychological Treatment Through the Conquerfear Study,” Supportive Care in Cancer 26, no. 12 (2018): 4207–4216, 10.1007/s00520-018-4294-y. 29882025

[13] M. K. Babadostu and A. Eyrenci , “Investigation of the Relationship Between Intolerance of Uncertainty, Metacognitions, Maladaptive Coping, and Fear of Cancer Recurrence Via Moderated Mediation Model,” Psycho‐Oncology 34, no. 1 (2025): e70076, 10.1002/pon.70076. 39799468

[14] L. Curran , L. Sharpe , C. MacCann , and P. Butow , “Testing a Model of Fear of Cancer Recurrence or Progression: The Central Role of Intrusions, Death Anxiety and Threat Appraisal,” Journal of Behavioral Medicine 43, no. 2 (2020): 225–236, 10.1007/s10865-019-00129-x. 31907743

[15] D. W. L. Ng , C. Foo , S. S. M. Ng , et al., “The Role of Metacognition and Its Indirect Effect Through Cognitive Attentional Syndrome on Fear of Cancer Recurrence Trajectories: A Longitudinal Study,” Psycho‐Oncology 29, no. 2 (2020): 271–279, 10.1002/pon.5234. 31663187

[16] J. E. Fardell , B. Thewes , J. Turner , et al., “Fear of Cancer Recurrence: A Theoretical Review and Novel Cognitive Processing Formulation,” Journal of Cancer Survivorship 10, no. 4 (2016): 663–673, 10.1007/s11764-015-0512-5. 26782171

[17] Y. Ruan , H. T. Reis , W. Zareba , and R. D. Lane , “Does Suppressing Negative Emotion Impair Subsequent Emotions? Two Experience Sampling Studies,” Motivation and Emotion 44, no. 3 (2020): 427–435, 10.1007/s11031-019-09774-w.

[18] R. N. Carleton , “Into the Unknown: A Review and Synthesis of Contemporary Models Involving Uncertainty,” Journal of Anxiety Disorders 39 (2016): 30–43, 10.1016/j.janxdis.2016.02.007. 26945765

[19] E. L. Gentes and A. M. Ruscio , “A meta‐analysis of the Relation of Intolerance of Uncertainty to Symptoms of Generalized Anxiety Disorder, Major Depressive Disorder, and obsessive‐compulsive Disorder,” Clinical Psychology Review 31, no. 6 (2011): 923–933, 10.1016/j.cpr.2011.05.001. 21664339

[20] P. M. McEvoy and A. E. Mahoney , “Intolerance of Uncertainty and Negative Metacognitive Beliefs as Transdiagnostic Mediators of Repetitive Negative Thinking in a Clinical Sample With Anxiety Disorders,” Journal of Anxiety Disorders 27, no. 2 (2013): 216–224, 10.1016/j.janxdis.2013.01.006. 23474912

[21] M. J. Dugas , M. Hedayati , A. Karavidas , K. Buhr , K. Francis , and N. A. Phillips , “Intolerance of Uncertainty and Information Processing: Evidence of Biased Recall and Interpretations,” Cognitive Therapy and Research 29, no. 1 (2005): 57–70, 10.1007/s10608-005-1648-9.

[22] Z. Shen , L. Zhang , S. Shi , C. Ruan , L. Dan , and C. Li , “The Relationship Between Uncertainty and Fear of Disease Progression Among Newly Diagnosed Cancer Patients: The Mediating Role of Intolerance of Uncertainty,” BMC Psychiatry 24, no. 1 (2024): 756, 10.1186/s12888-024-06201-4. 39482640 PMC11526518

[23] M. Blom , O. R. Guicherit , and M. T. Hoogwegt , “Perfectionism, Intolerance of Uncertainty and Coping in Relation to Fear of Cancer Recurrence in Breast Cancer Patients,” Psycho‐Oncology 32, no. 4 (2023): 581–588, 10.1002/pon.6102. 36702980

[24] P. Waroquier , F. Delevallez , D. Razavi , and I. Merckaert , “Psychological Factors Associated With Clinical Fear of Cancer Recurrence in Breast Cancer Patients in the Early Survivorship Period,” Psycho‐Oncology 31, no. 11 (2022): 1877–1885, 10.1002/pon.5976. 35674194

[25] K. Yook , K. H. Kim , S. Y. Suh , and K. S. Lee , “Intolerance of Uncertainty, Worry, and Rumination in Major Depressive Disorder and Generalized Anxiety Disorder,” Journal of Anxiety Disorders 24, no. 6 (2010): 623–628, 10.1016/j.janxdis.2010.04.003. 20439149

[26] D. W. L. Ng , R. Fielding , C. Tsang , et al., “Study Protocol of ConquerFear‐HK: A Randomised Controlled Trial of a Metacognition‐Based, Manualised Intervention for Fear of Cancer Recurrence Among Chinese Cancer Survivors,” BMJ Open 13, no. 1 (2023): e065075, 10.1136/bmjopen-2022-065075. PMC9872480 36669845

[27] D. W. L. Ng , “ConquerFear‐HK: A Randomised Controlled Trial of a metacognition‐based, Manualised Intervention for Fear of Cancer Recurrence Among Chinese Cancer Survivors,” Psychotherapy and Psychosomatics (2025). 10.1136/bmjopen-2022-065075 PMC9872480 36669845

[28] S. Simard and J. Savard , “Fear of Cancer Recurrence Inventory: Development and Initial Validation of a Multidimensional Measure of Fear of Cancer Recurrence,” Supportive Care in Cancer 17, no. 3 (2009): 241–251, 10.1007/s00520-008-0444-y. 18414902

[29] R. N. Carleton , M. A. P. J. Norton , and G. J. G. Asmundson , “Fearing the Unknown: A Short Version of the Intolerance of Uncertainty Scale,” Journal of Anxiety Disorders 21, no. 1 (2007): 105–117, 10.1016/j.janxdis.2006.03.014. 16647833

[30] H. Nordahl and A. Wells , “Measuring the Cognitive Attentional Syndrome Associated With Emotional Distress: Psychometric Properties of the CAS‐1,” International Journal of Cognitive Therapy 12, no. 4 (2019): 292–306, 10.1007/s41811-019-00056-4.

[31] N. Shrestha , “Detecting Multicollinearity in Regression Analysis,” American Journal of Applied Mathematics and Statistics 8, no. 2 (2020): 39–42, 10.12691/ajams-8-2-1.

[32] A. F. Hayes , T. D. Little , “Introduction to Mediation, Moderation, and Conditional Process Analysis : A Regression‐Based Approach,” in Methodology in the Social Sciences. 3rd ed. (Guilford Press, 2022).

[33] X. Sun , C. Zhu , and S. H. W. So , “Dysfunctional Metacognition Across Psychopathologies: A Meta‐Analytic Review,” European Psychiatry 45 (2017): 139–153, 10.1016/j.eurpsy.2017.05.029. 28763680

[34] A. Thingbak , L. Capobianco , A. Wells , and M. S. O'Toole , “Relationships Between Metacognitive Beliefs and Anxiety and Depression in Children and Adolescents: A Meta‐Analysis,” Journal of Affective Disorders 361 (2024): 36–50, 10.1016/j.jad.2024.05.123. 38815761

[35] N. M. Tauber , M. S. O’Toole , A. Dinkel , et al., “Effect of Psychological Intervention on Fear of Cancer Recurrence: A Systematic Review and Meta‐Analysis,” Journal of Clinical Oncology 37, no. 31 (2019): 2899–2915, 10.1200/jco.19.00572. 31532725 PMC6823887

