# Supplementary material for: The Role of Metacognition, Intolerance of Uncertainty, and Cognitive‐Attentional Syndrome in Differentiating Fear of Cancer Recurrence
Source: Psychooncology. 2026 Apr 29;35:e70475. doi: 10.1002/pon.70475 (PMC13127241; doi:10.1002/pon.70475)
Supplement: Supplementary file 1 — Figure S1: (A) Model showing a mediation role of CAS metacognitive strategies in the relationship between MCQ‐30 negative beliefs about worry and FCR status (non‐clinical FCR Vs subclinical/clinical‐significant FCR). (B) Model showing a mediation role of CAS metacognitive strategies in the relationship between MCQ‐30 need to control thoughts and FCR status (non‐clinical FCR Vs subclinical/clinical‐significant FCR). (C) Model showing a mediation role of CAS metacognitive strategies in the relationship between MCQ‐30 cognitive confidence and FCR status (non‐clinical FCR Vs subclinical/clinical‐significant FCR). (D) Model showing a mediation role of CAS metacognitive strategies in the relationship between Intolerance of uncertainty (IU) and FCR status (non‐clinical FCR Vs subclinical/clinical‐significant FCR). [file PON-35-e70475-s001.docx]

Supplementary Figure 1A. Model showing a mediation role of CAS metacognitive strategies in the relationship between MCQ-30 negative beliefs about worry and FCR status (non-clinical FCR Vs subclinical/clinical-significant FCR)

0.290** (without mediation)

0.428** (with mediation)

1.752**

0.080**

CAS metacognitive strategies

FCR status

MCQ-30 negative beliefs about worry

*p<0.05; ** p<0.001

Supplementary Figure 1B. Model showing a mediation role of CAS metacognitive strategies in the relationship between MCQ-30 need to control thoughts and FCR status (non-clinical FCR Vs subclinical/clinical-significant FCR)

-0.102* (without mediation)

-0.046 (with mediation)

1.425 **

0.143**

CAS metacognitive strategies

FCR status

MCQ-30 need to control thoughts

*p<0.05; ** p<0.001

Supplementary Figure 1C. Model showing a mediation role of CAS metacognitive strategies in the relationship between MCQ-30 cognitive confidence and FCR status (non-clinical FCR Vs subclinical/clinical-significant FCR)

0.066** (with mediation)

0.123** (without mediation)

0.084* (without mediation)

0.114* (with mediation)

1.131 **

0.123**

CAS metacognitive strategies

FCR status

MCQ-30 cognitive confidence

*p<0.05; ** p<0.001

0.746 **

0.104**

CAS metacognitive strategies

Supplementary Figure 1D. Model showing a mediation role of CAS metacognitive strategies in the relationship between Intolerance of uncertainty (IU) and FCR status (non-clinical FCR Vs subclinical/clinical-significant FCR)

IU

FCR status

*p<0.05; ** p<0.001
